# Genome-Wide and Exome-Capturing Sequencing of a Gamma-Ray-Induced Mutant Reveals Biased Variations in Common Wheat

**DOI:** 10.3389/fpls.2021.793496

**Published:** 2022-01-12

**Authors:** Yuting Li, Hongchun Xiong, Jiazi Zhang, Huijun Guo, Chunyun Zhou, Yongdun Xie, Linshu Zhao, Jiayu Gu, Shirong Zhao, Yuping Ding, Zhengwu Fang, Luxiang Liu

**Affiliations:** ^1^Hubei Collaborative Innovation Center for Grain Industry, College of Agriculture, Yangtze University, Jingzhou, China; ^2^National Engineering Laboratory of Crop Molecular Breeding/National Center of Space Mutagenesis for Crop Improvement, Institute of Crop Sciences, Chinese Academy of Agricultural Sciences, Beijing, China

**Keywords:** gamma-ray, genomic variation, exome, metabolic pathways, wheat

## Abstract

Induced mutagenesis is a powerful approach for the creation of novel germplasm and the improvement of agronomic traits. The evaluation of mutagenic effects and functional variations in crops is needed for breeding mutant strains. To investigate the mutagenic effects of gamma-ray irradiation in wheat, this study characterized genomic variations of wheat early heading mutant (*eh1*) as compared to wild-type (WT) Zhongyuan 9 (ZY9). Whole-genome resequencing of *eh1* and ZY9 produced 737.7 Gb sequencing data and identified a total of 23,537,117 homozygous single nucleotide polymorphism (SNP) and 1,608,468 Indel. Analysis of SNP distribution across the chromosome suggests that mutation hotspots existed in certain chromosomal regions. Among the three subgenomes, the variation frequency in subgenome D was significantly lower than in subgenomes A and B. A total of 27.8 Gb data were obtained by exome-capturing sequencing, while 217,948 SNP and 13,554 Indel were identified. Variation annotation in the gene-coding sequences demonstrated that 5.0% of the SNP and 5.3% of the Indel were functionally important. Characterization of exomic variations in 12 additional gamma-ray-induced mutant lines further provided additional insights into the mutagenic effects of this approach. Gene ontology (GO) and Kyoto Encyclopedia of Genes and Genome (KEGG) analysis suggested that genes with functional variations were enriched in several metabolic pathways, including plant–pathogen interactions and ADP binding. Kompetitive allele-specific PCR (KASP) genotyping with selected SNP within functional genes indicated that 85.7% of the SNPs were polymorphic between the *eh1* and wild type. This study provides a basic understanding of the mechanism behind gamma-ray irradiation in hexaploid wheat.

## Introduction

Induced mutagenesis is an effective method of generating novel genetic resources and improving important agronomic traits. Over the past few decades, induced mutagenesis has played a vital role in mining allelic variations from functional genes and breeding crop cultivars (Chaudhary et al., 2019; Lal et al., 2020). Genetic diversity of germplasm resources is the first step in cultivating new, high-yield crop cultivars (Fu, 2015). However, the genetic breeding pool is limited due to widely planted elite cultivars that harbored homozygous favorable alleles (Bhalla, 2006; Ribera et al., 2020). Therefore, it is necessary to increase genetic diversity and enrich new allelic polymorphisms to sustainably breed crops through induced mutagenesis.

Approaches commonly used for plant mutagenesis include physical (e.g., gamma-ray and ion beams) and chemical (e.g., ethyl methanesulfonate, EMS) factors. There are several genomic mutation characteristics caused by these mutagenic factors. The chemical mutagen EMS is prone to interact with the phosphate, pyrimidine, and purine groups within the nucleotide and induce point mutations (Gary, 1984). However, physical rays carrying high energy are capable of breaking double-stranded DNA and can introduce small fragment insertions and deletions (Indel) or structural variations in chromosomes (e.g., inversions and translocations) (Ikehata and Ono, 2011; Chatterjee and Walker, 2017). Polyploid crops can tolerate higher doses of mutagenic treatments than diploids due to the redundant effect of homoeologous copies, which generate a relatively high density of mutations (Wang et al., 2012; Uauy et al., 2017). The gamma-ray, one of the traditional physical mutagens, has been widely utilized in crop breeding and improvement of ornamental plants. In rice, numerous valuable agronomic traits, including yield (Kato et al., 2020), plant height (Andrew et al., 2021), and abiotic stress (Abdelnour-Esquivel et al., 2020), have been identified in gamma-ray-induced mutants. Moreover, this technique contributed to the creation of novel genetic resources and increased genetic diversity for wheat breeding (Hussain et al., 2021). In addition to its application in cereal corps, gamma-ray irradiation can effectively diversify morphological variations in ornamental plants such as Ruscus (Kutsher et al., 2020), *Gypsophila paniculate* (Li F. et al., 2020), and Tuberose (Kutty et al., 2019).

Although the biological effects induced by mutagenesis in plants have been widely reported, the molecular basis of mutagenesis requires further study. Recent advancements in high-throughput sequencing technology have facilitated the investigation of variation patterns and mutation characteristics resulting from induced mutagenesis in various plants. In tomato plants, more than 98% of EMS-induced mutations were single-nucleotide polymorphisms such as transitions or transversions, whereas in the mutant irradiated with gamma-ray, the proportion of single nucleotide polymorphism (SNP) decreased and the number of Indel mutations increased (Shirasawa et al., 2016). This suggests that EMS and gamma-ray treatment have divergent effects on mutation types in plants. Additionally, a biased proportion of the type of single-base mutation was observed in different crops. A study on the rice cultivar “Gaogengnuo” suggested that the number of transitions was significantly higher than transversions under different doses of gamma-ray irradiation (Zhang et al., 2020). Similar results were observed in the spaceflight-induced mutant *st1* in wheat (Xiong et al., 2017).

Previous studies have assessed the mechanisms of chemical and physical mutagenesis primarily based on model plants such as *Arabidopsis* and rice (Cheng et al., 2014; Jo and Kim, 2019; Bhuvaneswari et al., 2020; Zhang et al., 2020; Andrew et al., 2021). However, mutation characteristics in wheat remain poorly understood at the genome-wide level, partially due to its large genome size and high-repetitive sequences (International Wheat Genome Sequencing Consortium [IWGSC], 2018). Traditional mutation screening such as TILLING is an effective strategy to identify point mutation but lacks the power to detect other types of variations (Uauy et al., 2009). The recent release of high-quality reference genomes has made it possible to investigate the mutagenic effects of physical irradiation through cost-effective next-generation sequencing technology in polyploid wheat. In this study, we identified SNP and Indel variations from a series of gamma-ray-induced mutant lines *via* genome resequencing or exome-capturing sequencing. Variation patterns and mutation hotspot regions were characterized on multiple chromosomes, whereas GO and KEGG enrichment analysis of genes with functional variations identified key pathways involved in plant–pathogen interaction and ADP binding. This study provides a basic understanding of genome-wide variations induced by gamma-ray and the molecular basis for mutation breeding in common wheat.

## Materials and Methods

### Plant Materials

The dry seeds of the inbred wheat line Zhongyuan 9 (ZY9, wild type) were treated with gamma-rays at a dosage of 284 Gy. After the treatment, the seeds were planted at the Zhongpuchang station of the Institute of Crop Sciences, Chinese Academy of Agricultural Sciences (Beijing, China). The mutants were continuously planted to M_7_ generation after which a stably inherited mutant with an early heading date phenotype was identified, which was designated as *early heading 1* (*eh1*) (Li Y. et al., 2020). A total of 12 mutant lines (≥M_6_ generation) derived from the Chinese winter cultivar Jing411 by gamma-ray irradiation at a dosage of 250 Gy were used for exome-capturing sequencing.

### Isolation of Genomic DNA

The fresh leaves were sampled from the *eh1* and wild-type (WT) at the seedling stage. Genomic DNA was extracted according to the PVP-40 method (Guillemaut and Mardchal-Drouard, 1992). The quantity and quality of DNA were determined using a NanoDrop ND-2000 Spectrophotometer (Thermo Fisher Scientific, United States) and agarose gel electrophoresis. The final DNA was diluted to 200 ng μl^–1^ with TE buffer and stored at –80^°^C.

### Whole-Genome Resequencing and Quality Control

To eliminate the minimal variations derived from individuals, we mixed eight DNA samples from *eh1* or WT for library construction. The genomic DNA was randomly interrupted by ultrasonic fragmentation and detected by agarose gel electrophoresis to recover approximately 350-bp DNA fragments. The interrupted DNA fragments were combined with connector primers to construct a sequencing library. The genomic library was sequenced on an Illumina HiSeq 2500 machine by Annoroad Gene Technology Co., Ltd. (Beijing China). The resulting image data were converted to raw sequencing data by Illumina bcl2fastq2 (v2.7). To obtain clean reads, low-quality raw reads were removed based on three criteria: reads containing more than five adapter-contaminated bases; reads with the number of low-quality bases (Phred quality score < 19) accounting for more than half of total bases; and reads with more than 5% unidentified bases.

### Variant Calling and Annotation

The clean reads were mapped to the Chinese Spring reference genome (IWGSC RefSeq assembly v1.0) using the Burrows-Wheeler aligner (Li and Durbin, 2009). SAMtools v1.2 tools were used to sort reads (Li et al., 2009), and duplicate reads caused by PCR were discarded using Picard tools v1.13 MarkDuplicates.^1^ Reads mapped to multiple loci were also removed. Based on the alignment, the GATK software (McKenna et al., 2010) was used for calling putative SNP and Indel variations and for calculating the homozygous and heterozygous loci. To avoid misalignments and sequence errors in variant calling, unreliable SNP and Indel loci were discarded based on the following criteria: sequence depth lower than 4 or greater than 100, mapping quality less than 4 (MQ < 4), and a variant with more than 2 genotypes. All high-quality SNP and Indel variants were annotated using ANNOVAR software (Wang et al., 2010) based on the reference genome annotation (IWGSC RefSeq annotation v1.0).

### Library Preparation, Exome-Capturing Sequencing, and Variant Calling

The DNA bulks for *eh1* and WT were constructed by mixing 16 DNA samples equally. The genomic library was constructed using a KAPA HyperPlus Kit (cat number 7962380001), according to the manufacturer’s instructions. The length of exome-capturing probes ranged from 55 to 105 bp and covers approximately 200 Mb of the exonic regions in wheat. The probes were then added to the genomic library for liquid-phase hybridization, while streptavidin magnetic beads were added to the reaction solution to enrich biotin-tagged probes. After elution, the enriched DNA fragments were amplified by PCR to obtain exome-capturing sequencing libraries, which were then subjected to high-throughput sequencing on the NovaSeq 6000 platform (Illumina, San Diego, CA), according to the manufacturer’s instructions, by Bioacme Biotechnology Co., Ltd. (Wuhan, China). The Fastp software^2^ was used to filter out the low-quality and unidentified reads. The clean reads were aligned to the Chinese Spring reference genome^3^ and then sorted using the SAMtools^4^ to yield BAM files. Repetitive sequences caused by PCR were identified and filtered out using Biobambam2 software.^5^ The statistics of mapping data, including mapping rate, sequencing depth, and coverage rate, were analyzed using the Qualimap software.^6^ BCFtools (Li, 2011) were used to call potential SNP, and Indel loci and functional variations were annotated by ANNOVAR software (Wang et al., 2010).

### Gene Ontology and Kyoto Encyclopedia of Genes and Genome Enrichment Analysis

For genes with functional variations (frameshift or stop-gain/loss), GO terms were extracted based on the gene homology.^7^ Enrichment analysis was performed with the GOseq R package. Enriched GO terms with corrected *p*-value < 0.05 were considered significant. KEGG pathways for mutated genes were retrieved.^8^ The statistical significance of enriched KEGG pathways was tested using KOBAS software. All data were visualized with the ggplot R package.

### Development of Kompetitive Allele-Specific PCR Markers and Genotyping

A total of 26 SNPs were selected and converted to Kompetitive Allele-Specific PCR (KASP) markers. The 150-bp DNA sequence flanking SNP loci were retrieved to align to the reference genome. Primers used for KASP assay were designed with the Galaxy online tool.^9^ For the two forward primers, each was added with standard FAM or HEX complementary tails (FAM 5′-GAAGGTGACCAAGTTCATGCTT-3′ and HEX 5′-GAAGGTCGGAGTCAACGGATT-3′) at the 5′ end. All primers were synthesized by Sangon Biotech (Beijing, China). The PCR was performed as previously described (Li Y. et al., 2020). Fluorescence signals were collected using the FLUOstar Omega reader (BMG LABTECH, Germany), and genotyping was conducted with the Klustercaller software (v2.22.0.5, LGC Genomics).

## Results

### Genome Resequencing and Mapping

The Illumina high-throughput sequencing platform was used to obtain a total of 2,493,075,922 (373,961 Mb) and 2,546,130,554 (381,919 Mb) raw reads from the *eh1* and ZY9, respectively. After filtering out low-quality and adapter-contaminated reads, we obtained 2,439,545,586 (365,931 Mb) and 2,479,168,134 (371,875 Mb) clean reads from the *eh1* and ZY9, respectively. More than 360 Gb raw data were obtained for each sample. The Phred quality score Q30 (an error rate of 0.1%) of the clean reads was 94.2 and 94.3%, respectively (Supplementary Figure 1), indicating that the sequencing data were suitable for further analysis. The filtered high-quality reads were aligned to the Chinese Spring Reference Genome (IWGSC RefSeq assembly v1.0). After removing incorrect alignments and repetitive sequences, more than 98% of the clean reads were successfully mapped to the reference genome. The repetitive sequences caused by PCR account for 22.2 and 20.8% in the *eh1* and ZY9, respectively. Approximately 95% of the reference sequences were mapped at least once and 92% of them were mapped at least four times (Table 1). The average sequencing depth of the *eh1* and ZY9 samples was approximately 25X (Supplementary Figure 2).

**TABLE 1:** Statistics of whole genome re-sequencing and mapping data.

Sample	*eh1*	Wildtype
Number of raw reads	2,493,075,922	2,546,130,554
Number of clean reads	2,439,545,586	2,479,168,134
Number of clean bases	365,931,837,900	371,875,220,100
Mapped bases	359,893,553,535	364,529,898,773
Mapping rate (%)	98.35	98.02
Repetitive rate (%)	22.20	20.80
Coverage rate ≥ 1X (%)	95.16	95.43
Coverage rate ≥ 4X (%)	92.86	93.19
Average sequencing depth	25.21	25.53

### Identification and Distribution of Single Nucleotide Polymorphism and Indel

**FIGURE 1:**
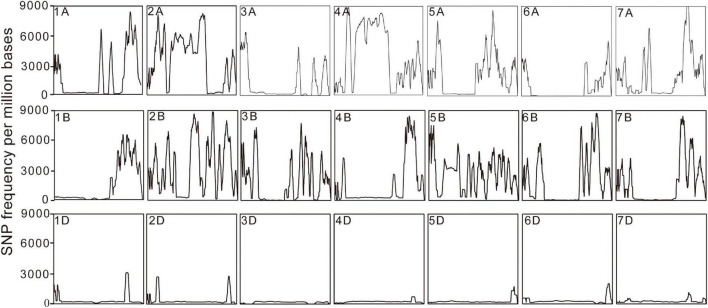
Diagrammatic distribution of SNP frequency across 21 chromosomes. The rectangle represents a distribution of SNP frequency in each chromosome. The *x*-axis indicates the physical position on the chromosomes and the *y*-axis shows the SNP number in every million bases.

Putative SNPs and Indel between the sample and the reference sequence were identified based on the read alignment. A total of 40,952,050 SNPs were identified in the *eh1* and 28.1% of them were heterozygous. To identify reliable polymorphic sites between the *eh1* and WT, heterozygous SNPs or SNPs with sequencing depth >100 or <4 were removed. In total, 23,537,117 homozygous SNPs with high confidence were chosen for downstream analysis. To analyze the distribution patterns of SNPs in the genome, the SNP number was counted on each chromosome. Results demonstrated that the SNP frequency varies among different chromosomes (Figure 1). A total of 120,000 SNPs were distributed on chromosome 4D, and more than 3 million SNPs were located on chromosome 4A (Table 2). Except for the difference of SNP frequency among each chromosome, the uneven distribution of SNPs was observed in the subgenomes A, B, and D. The average SNP frequency in the subgenome D is approximately 1/7 of that in subgenomes A and B (Table 2).

**TABLE 2:** Numbers and density of SNP and Indel variations on all chromosomes.

Chromosome	SNP numbers	SNP density (/Mb)	Indel numbers	Indel density (/Mb)
1A	1,078,430	1815.5	72,408	121.9
1B	1,027,394	1467.7	71,942	102.8
1D	204,414	403.2	23,188	45.7
2A	2,797,338	3581.7	154,887	198.3
2B	2,709,968	3333.3	178,616	219.7
2D	260,449	392.8	31,869	48.1
3A	814,340	1068.7	62,718	82.3
3B	1,590,668	1891.4	109,414	130.1
3D	125,267	199.8	15,836	25.3
4A	3,093,150	4091.5	174,294	230.5
4B	1,063,554	1552.6	68,982	100.7
4D	120,996	232.2	12,606	24.2
5A	1,460,181	2025.2	97,871	135.7
5B	1,874,889	2586.1	126,961	175.1
5D	155,776	270.0	19,419	33.7
6A	510,962	811.1	48,492	77.0
6B	1,429,256	1952.5	94,927	129.7
6D	172,587	355.8	20,017	41.3
7A	1,574,567	2105.0	110,313	147.5
7B	1,275,568	1674.0	89,764	117.8
7D	197,363	303.6	23,944	36.8
A genome	11,328,968	2214.1	720,983	141.9
B genome	10,971,297	2065.3	740,606	139.4
D genome	1,236,852	308.2	146,879	36.4
Whole genome	23,537,117	1529.2	1,608,468	105.9

### Variation Patterns of Single Nucleotide Polymorphism and Indel

**FIGURE 2:**
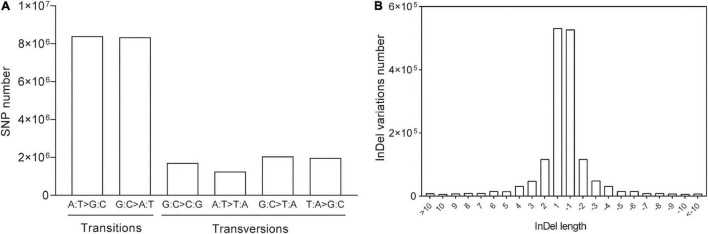
Variation patterns of genomic SNPs and Indels in *eh1*. **(A)** Numbers of specific types of SNPs in the whole genome of the gamma-ray-induced mutant *eh1*. **(B)** Statistics of Indel size identified by genome resequencing. The positive and negative values represent insertions and deletions, respectively.

To investigate the variation patterns between the *eh1* mutant and WT, SNP types and Indel length were analyzed. The results demonstrated that 70.56% of the SNPs were transitional, of which 35.40% were A → G or T → C and 35.16% were G → A or C → T. However, the proportion of transversions (C/T → A/G or A/G → C/T) accounted for 29.44% of the total, suggesting that the frequency of transitions is higher than that of transversions (Figure 2A). For Indel, the small fragment insertions and deletions were 50.18 and 49.88%, respectively. The statistical analysis of the length of Indel demonstrated that over 90% of the Indel ranged from 1 to 10 bp, and 61.25% of them were single-base insertion or deletions (Figure 2B).

### Exomic Variation Patterns Induced by Gamma-Ray

To characterize the variations of the *eh1* mutant at the exomic level, the genomic DNA was hybrid with exome-capturing probes for high-throughput sequencing. A total of 13.6 and 14.2 Gb sequencing data were obtained in the *eh1* and ZY9, with an average sequencing depth of 69.5 X and 72.5 X, respectively. After removing the adaptor-contained sequences and the low-quality bases, more than 1,358 million reliable bases (1,358 Mb) were obtained in each sample. The clean reads were aligned to the reference genome, and over 99.8% of them were successfully matched. Based on the alignment, approximately 73% of the target region was sequenced at least 5 times, whereas 43% of them with a sequencing depth of more than 30 X (Table 3). For *eh1*, a total of 1,305,486 SNP and 105,260 Indel variations in the exome were detected, and the heterozygous rate was 32.15 and 40.38%, respectively. After filtering out the same loci that were detected both in the *eh1* and the WT, a total of 217,948 homozygous SNPs and 13,554 Indels were identified to be presented between the two samples.

**TABLE 3:** Statistics of exome-capturing sequencing and mapping data.

Sample	Mutant	Wildtype
Number of raw reads	92,668,310	94,511,490
Number of clean reads	91,693,900	93,029,978
Number of clean bases	13,582,499,408	14,176,723,500
Mapping rate (%)	99.88	99.89
Coverage rate ≥ 5X (%)	74.66	73.76
Coverage rate ≥ 20X (%)	54.82	54.28
Coverage rate ≥ 30X (%)	44.06	43.68
Average sequencing depth (%)	69.50	72.54

**FIGURE 3:**
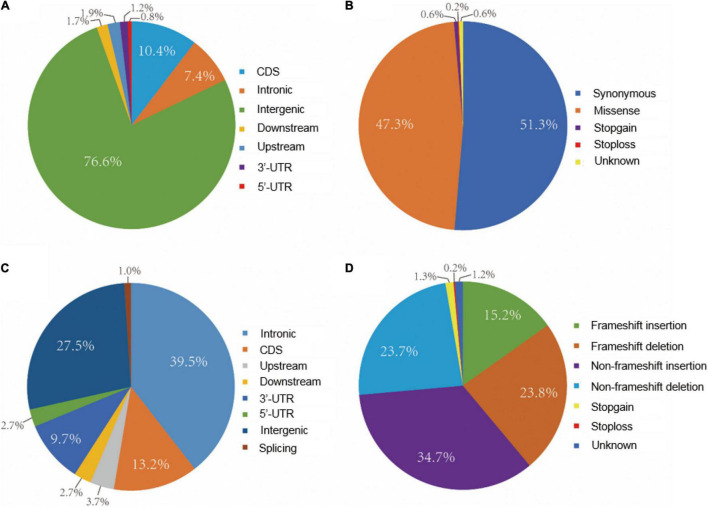
Distribution and functional annotation of exomic variations between *eh1* and WT. **(A)** Frequency of SNPs in different exome regions. **(B)** Functional annotation of SNPs in gene-coding region. **(C)** Frequency of Indel in different regions in the exome. **(D)** Functional annotation of Indels in gene-coding region.

The distribution of SNPs and Indel among 21 chromosomes was analyzed based on homozygous variations between the *eh1* and WT. The results demonstrated that most variations were located on the distal part of chromosomes. However, a relatively even distribution of mutations was observed on chromosomes 1D, 2B, 3B, 4A, 6A, 6B, and 7A (Supplementary Figure 3). To further understand the variation patterns within structural genes, SNP and Indel variations were functionally annotated. Approximately 7.4 and 12.4% of the SNP were annotated on introns and exons, respectively. Moreover, several variations were detected on the intergenic region due to the high coverage of capturing probes (Figure 3A). Functional annotation of SNPs in the gene-coding sequence suggested that the synonymous and missense variations accounted for 51.3 and 47.3%, respectively. Additionally, 0.8% of the functional SNP were stop-gain/loss (Figure 3B). Indel annotations demonstrated that 72.5% of them were distributed on the genic region, in which 13.2 and 1.0% were located on coding and splicing regions, respectively (Figure 3C). Functional analysis of Indel suggested that the proportion of frameshift, non-frameshift, and stop-gain/loss accounted for 39.0, 58.4, and 1.5%, respectively (Figure 3D).

**FIGURE 4:**
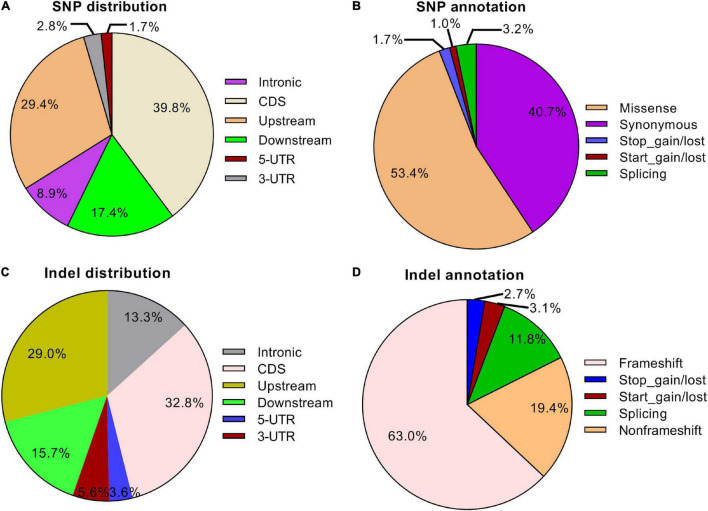
Distribution and functional annotation of exomic variations in 12 mutant lines. **(A)** Distribution of SNPs in different regions in the exome. **(B)** Functional annotation of SNPs in structural genes. **(C)** Distribution of Indels in different regions in the exome. **(D)** Functional annotation of Indels in structural genes.

To further investigate the effects of gamma-ray irradiation on the wheat exome, we sequenced 12 mutant lines (≥M_6_ generation) and characterized SNP and Indel variation patterns. A total of 1,496,999 homozygous SNPs were identified in all mutant lines, with an average of 124,748 loci in each line. We observed a biased distribution of SNP numbers in different subgenomes and chromosomes. The Chr. 4D contained a relatively small number of SNPs whereas the highest amount of SNPs was identified on Chr. 2B (Supplementary Table 1). Moreover, the SNP frequency on the D subgenome was notably lower compared with the subgenomes A and B (Supplementary Figure 4), which is consistent with the SNP distribution in *eh1*. This indicates that gamma-ray irradiation is sensitive in different genomic regions. Additionally, more than half of the SNP were transitions, in which the A:T to G:C or G:C to A:T types showed minimal preference (Supplementary Figure 5A). Similar to the Indel variations in the *eh1* among 138,695 Indel variations identified in 12 mutant lines, the frequency of fragment insertions was comparable to that of deletions, whereas the number of Indels decreased with the increment of fragment size (Supplementary Figure 5B). To explore the functional variations resulting from gamma-ray irradiation, we analyzed the SNP and Indel distribution within different gene structures. Approximately 30–40% of variations were located on gene-coding sequence (CDS) and variations identified in the untranslated region accounted for less than 10% (Figures 4A,C). Due to the high coverage rate of exome-capturing probes, a certain number of SNPs and Indels were annotated on gene up- and down-stream or intergenic regions. Variations in these regions were excluded from further analysis. Functional annotation of CDS variations suggested that over half of the SNPs altered corresponding amino acids and approximately 6% were start or stop codon or splicing variants (Figure 4B). In contrast, frameshift variations accounted for 63% of Indels, indicating that the proportion of functionally important variations of Indel was relatively higher than that of SNP (Figure 4D). These results indicate that the majority of the Indels disrupted gene function. Altogether, the genomic characterization of *eh1* and exomic analysis of multiple mutant lines suggested that the D subgenome shows higher tolerance to gamma-ray irradiation, in which chromosome 4D represents the most insensitivity compared with others. Functional annotation indicated that about half of the induced mutations in the gene-coding sequence are disruptive to primary proteins.

### Gene Ontology and Kyoto Encyclopedia of Genes and Genome Enrichment Analysis

**FIGURE 5:**
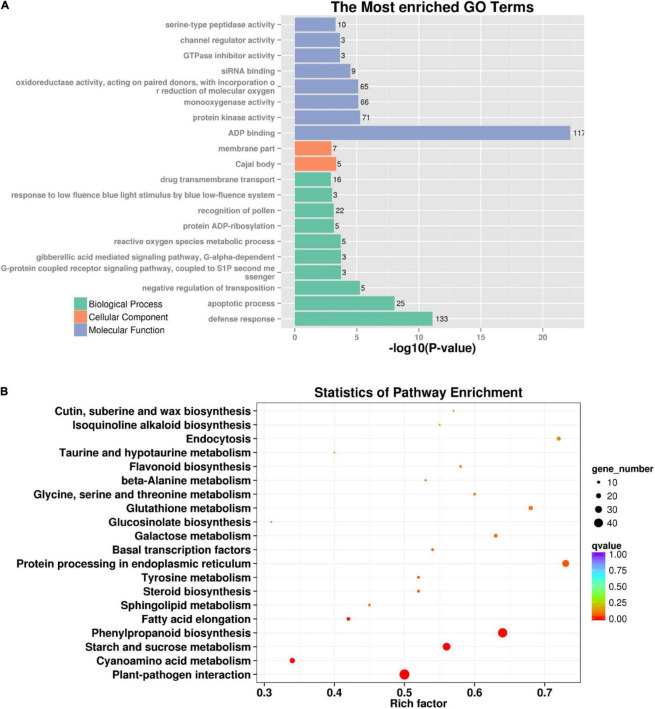
GO and KEGG enrichment analysis of genes with functional variations. **(A)** Top 20 GO terms are enriched in genes with termination or frameshift variations. The significance of enriched terms was determined by REVIGO. **(B)** The most enriched KEGG pathways in genes with termination or frameshift variations. The rich factor represents the degree of mutated genes in a particular pathway. The number of enriched genes is indicated by the size of the circle, and the circle color reflects the range of the corrected *p-*value.

To investigate the mutagenic effects of gamma-ray irradiation on metabolic pathways, 889 genes with functionally important variations were used to perform GO and KEGG enrichment analyses. GO analysis demonstrated that both the biological process and molecular function of mutated genes were enriched, including ADP binding, protein kinase activity, defense response, and apoptotic processes (Figure 5A). The pathways primarily enriched by KEGG analysis were plant–pathogen interactions, starch and sucrose metabolism, phenylpropanoid biosynthesis, and protein processing in the endoplasmic reticulum (Figure 5B). Moreover, we identified functional variations in 12 mutant lines. In total, 1,466 genes harboring functional variations were identified in two or more mutant lines. GO analysis indicated that most of these genes were involved in biological processes and molecular function, including ADP binding and defense response (Supplementary Figure 6A). Additionally, KEGG analysis identified flavonoid biosynthesis and plant–pathogen interactions as the most significantly enriched pathways (Supplementary Figure 6B). Altogether, GO and KEGG analysis by mutated genes from *eh1* and 12 mutant lines identified overlapped metabolic pathways, indicating that these pathways are sensitive to gamma-ray irradiation.

### Validation of Selected Single Nucleotide Polymorphism

**FIGURE 6:**
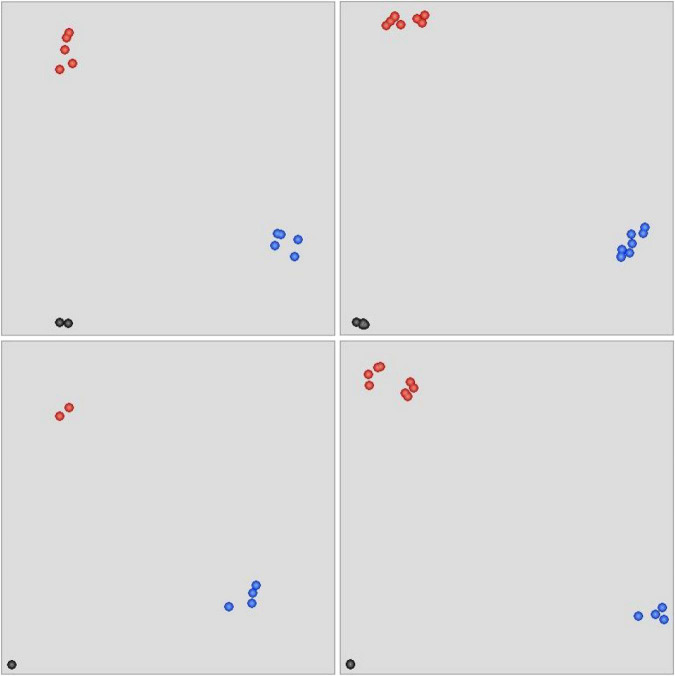
Scatter plot of KASP assay for genotyping selected SNPs. The blue-colored dots represent alleles with FAM tails. The HEX-tailed allele is indicated by red dots. Black dots reflect the negative control or alleles yielding no PCR products. All 26 SNPs were genotyped with four KASP assays.

To test the authenticity of variations between the *eh1* and WT, a total of 26 putative SNPs distributed in 10 genes involved in important agronomic traits were selected to develop KASP markers. These markers were used to analyze the genotype of *eh1* and WT. Fluorescence signals were detected in 21 of 26 markers (Supplementary Table 2). Of these markers, 18 (85.7%) were polymorphic between the *eh1* and WT (Figure 6), suggesting that the SNPs identified by high-throughput sequencing were reliable.

### Major Flowering Gene Variations Between *eh1* and Wild-Type

We previously identified a large fragment deletion in the vernalization gene *Vrn-B1* responsible for heading date variation in a recombinant inbred population derived from *eh1* and LX987. However, sequence comparison among *eh1*, WT, and LX987 demonstrated that this variation has resulted from the genetic background in parent lines (Li Y. et al., 2020). To investigate the potential causal variants for the early heading phenotype of *eh1*, we identified and annotated SNP and Indel variations within the vernalization and photoperiod genes. A total of 16 SNPs and 1 Indel were identified in *Vrn-A1*, *Vrn-B2*, *Vrn-D3*, and *Ppd-2A*. Functional annotation suggested that 8 out of 11 exonic SNPs were non-synonymous variants. Notably, a single-base insertion was identified in the exonic region of *Vrn-D3* in the *eh1* (Supplementary Table 3). Further validation and association analysis are needed to verify whether these mutations resulted in the early heading phenotype of the *eh1* mutant.

## Discussion

Common wheat is one of the most widely grown crops in the world, which provides approximately 20% of all calories consumed by humans (International Wheat Genome Sequencing Consortium [IWGSC], 2018). The induced mutation technique has been widely used in crop germplasm creation and breeding new varieties (Parry et al., 2009; Chaudhary et al., 2019). Due to the allohexaploid characteristics and the high-repetitive genome sequence, genomic research in common wheat has lagged behind other cereal crops (e.g., maize and rice). There are relatively few reports that assess the characteristics and patterns of wheat mutagenesis caused by various induced factors at the genome-wide level (Renny-Byfield and Wendel, 2014; Uauy, 2017). As such, exploring characteristics of the wheat genome induced by mutagenesis technology will provide a basic reference for future studies assessing the mutation mechanism of this complex crop and will help inform mutation breeding. In this study, the gamma-ray-irradiated mutant *eh1* and wild type were resequenced to explore the signatures and patterns of the genomic variations. A total of 737.7 Gb data with an average sequencing depth of 25 X were obtained, of which more than 98% of clean reads were mapped to the reference genome. This suggests the sequencing data were reliable and suitable for further analysis. The distribution of SNPs and Indels among different chromosomes suggested that the variation frequency in D subgenomes was significantly lower than in the subgenomes A and B. Due to two natural hybridization events that occurred in the formation of hexaploid wheat, the genetic diversity of different subgenomes differed, whereas the genetic plasticity of the D genome was lower than that of the subgenomes A and B (Dubcovsky and Dvorak, 2007). This indicates that the subgenome D may have a relatively high tolerance to mutagenesis, leading to a low variation frequency. In addition to differences between subgenomes, mutations were not distributed in different chromosomal regions. Statistical analysis of SNP distribution on 21 chromosomes demonstrated that the SNP frequency in some regions was significantly higher than in other chromosomal regions (Figure 1), indicating the presence of “mutation hotspots.” Similarly, transcriptome sequencing with mutants induced by EMS, gamma-ray, and ion beams also identified regions with a high frequency of mutations at specific locations of certain chromosomes (Xiong et al., 2020a,b), indicating that the sensitivity of some chromosome segments to mutagenesis in wheat varies greatly. In tomato plants, EMS treatment was more likely to produce C/G to T/A transitions (Shirasawa et al., 2016). Moreover, the number of transitions in spaceflight-induced wheat is approximately two times higher than that of transversions (Xiong et al., 2017). This study presented a genome-wide analysis of SNPs in the gamma-ray-induced wheat mutant and showed that the frequency of transition was significantly higher than that of a transversion and that during transitions, there was no significant difference in the number of SNPs from pyrimidine to pyrimidine or purine to purine (Figure 2A). These results indicate that the same mutagenic treatment may give rise to different variation patterns in crops. Chemical mutagens such as EMS typically produce point mutations due to their specific interaction with the subcomponent in DNA bases (Gary, 1984). Physical rays and ion beams are powerful enough to break DNA double-strands and introduce fragment insertions, deletions, and other structural variations (Henner et al., 1982; Ikehata and Ono, 2011; Chatterjee and Walker, 2017). In the mutant *eh1*, genome resequencing identified a large number of SNPs. Similarly, whole-genome resequencing identified extensive single-base substitutions and a relatively small number of structural variations in gamma-ray, carbon(C)-ion beams, and fast-neutron-irradiated rice mutants, suggesting that these physical treatments are efficient in inducing SNP variations (Cheng et al., 2014; Li et al., 2016, 2019). Analysis of the *eh1* and ZY9 showed that the majority of Indels were single-base insertions or deletions and that the Indel frequency gradually decreased with the increment of fragment size (Figure 2B). Accurately detecting large Indels from genome resequencing data relies on the reference genome and sequencing depth, which means that there are limited Indel variations in large fragments from the complex wheat genome that have been identified.

Compared with genome resequencing, exome-capturing sequencing is cost-effective and can rapidly analyze the gene-coding sequences. Therefore, it is widely used in crops with large genomes (Kaur and Gaikwad, 2017). In tetraploid and hexaploid wheat, over 2,000 EMS-induced mutant lines were analyzed through exome-capturing sequencing and provided an abundance of functional mutations in more than 90% of wheat genes (Krasileva et al., 2017). The exome-capturing sequencing was conducted to explore mutation characteristics in the exonic region. The SNP and Indel in the exome were unevenly distributed, and several of them were concentrated in terminal chromosomal regions (Supplementary Figure 3), which was correlated with the distribution of gene density in chromosomes. The SNP frequency in gene-coding regions exceeded in non-coding regions, whereas the number of functional and non-functional mutations did not differ. However, the Indel frequency in gene-coding regions was lower than in non-coding regions. Similar variation patterns were observed in a gamma-ray-induced rice mutant (Zhang et al., 2020). These results indicate that the SNP and Indel variation patterns in gene regions caused by induced mutations differed. Moreover, variation characterization and the annotation of 12 gamma-ray-induced mutant lines in the exome showed similar mutagenic effects with the *eh1*, though the average SNP and Indel frequency in the 12 mutant lines were slightly lower than *eh1*. Since the *eh1* mutant was planted seven generations before identification and treated with a relatively high dosage of irradiation, more spontaneous variations and a strong irradiative effect could lead to a high variation frequency. To investigate the mutagenic effects of gamma-rays on metabolic pathways, GO and KEGG enrichment analyses were performed with functionally mutated genes in *eh1* and 12 additional mutant lines. Several pathways were significantly enriched, including plant–pathogen interaction and ADP binding. Similarly, pathway enrichment analysis with differentially expressed genes in gamma-ray, and EMS-induced mutants identified overlapped metabolic pathways in wheat (Xiong et al., 2020b), suggesting that these pathways are sensitive to induced mutagenesis.

The rate of spontaneous and induced mutation in *Arabidopsis* is approximately 7 × 10^–9^/bp and 2–6 × 10^–7^/bp, respectively (Jo and Kim, 2019). The mutation rate is also dependent on genome size and genetic composition in various plants. In EMS-induced mutants, the mutation rate ranged from 1/100 to 1/1,000 kb (Sikora et al., 2011). Mutation frequency typically increases as mutagen dosage increases. Polyploid crops are more tolerant of mutagenesis than diploids due to their homoeologous copies (Uauy et al., 2017). The mutation rate of SNPs in hexaploid wheat can reach 1/23–1/40 kb (Slade et al., 2005; Dong et al., 2009; Uauy et al., 2009). In this study, more than 20 million SNPs were identified by genome resequencing and exome-capturing sequencing in the gamma-ray-induced mutant and the wild type. One limitation of the analysis is that genome-wide analysis was performed on only two samples and that spontaneous mutations and inherent polymorphic sites could not be excluded from analysis and thus produced a relatively high frequency of variation. To better understand the molecular basis of mutagenesis in wheat, advanced sequencing technology and multiple samples are needed to eliminate background noise.

## Conclusion

We identified SNP and small fragment insertions or deletions induced by gamma-ray irradiation in the wheat mutant *eh1* and 12 additional mutant lines by genome resequencing or exome-capturing sequencing. The variation frequency in the subgenome D was remarkably lower than that of subgenomes A and B. The distribution of SNP frequency fluctuates on the position of each chromosome and the variation “hotspot” exists in certain regions. In gene coding sequences, approximately half of the SNPs and Indels are disruptive to putative protein function. The creation of functional variations in *eh1* and 12 additional mutant lines provided genetic resources for gene functional study and agronomic trait improvement. The characterization of genomic variation features and patterns supplies a basis for understanding the mechanism of mutagenesis and mutation breeding in wheat.

## Data Availability Statement

The datasets presented in this study can be found in online repositories. The names of the repository/repositories and accession number(s) can be found below: https://www.ncbi.nlm.nih.gov/bioproject/PRJNA771357/.

## Author Contributions

LL and ZF conceived the project and revised the manuscript. YL and HX conducted most of the experiments. HG, JZ, and CZ participated in the data analyses and visualization. YX, JG, LZ, SZ, and YD assisted in the enrichment analyses and KASP assays. YL wrote the first draft of the manuscript. All authors have read and approved the final manuscript.

## Conflict of Interest

The authors declare that the research was conducted in the absence of any commercial or financial relationships that could be construed as a potential conflict of interest.

## Publisher’s Note

All claims expressed in this article are solely those of the authors and do not necessarily represent those of their affiliated organizations, or those of the publisher, the editors and the reviewers. Any product that may be evaluated in this article, or claim that may be made by its manufacturer, is not guaranteed or endorsed by the publisher.

## Footnotes

1
http://broadinstitute.github.io/picard/

2
https://github.com/OpenGene/fastp

3
https://www.wheatgenome.org/

4
http://www.htslib.org/

5
https://www.sanger.ac.uk/science/tools/biobambam

6
http://qualimap.conesalab.org/

7
http://www.geneontology.org

8
http://www.genome.jp/kegg/

9
https://galaxy.triticeaetoolbox.org/
