# Supplementary material for: Genome-Wide and Exome-Capturing Sequencing of a Gamma-Ray-Induced Mutant Reveals Biased Variations in Common Wheat
Source: Front Plant Sci. 2022 Jan 12;12:793496. doi: 10.3389/fpls.2021.793496 (PMC8790116; doi:10.3389/fpls.2021.793496)
Supplement: Supplementary file 1 [file Data_Sheet_1.docx]

Supplementary Figures


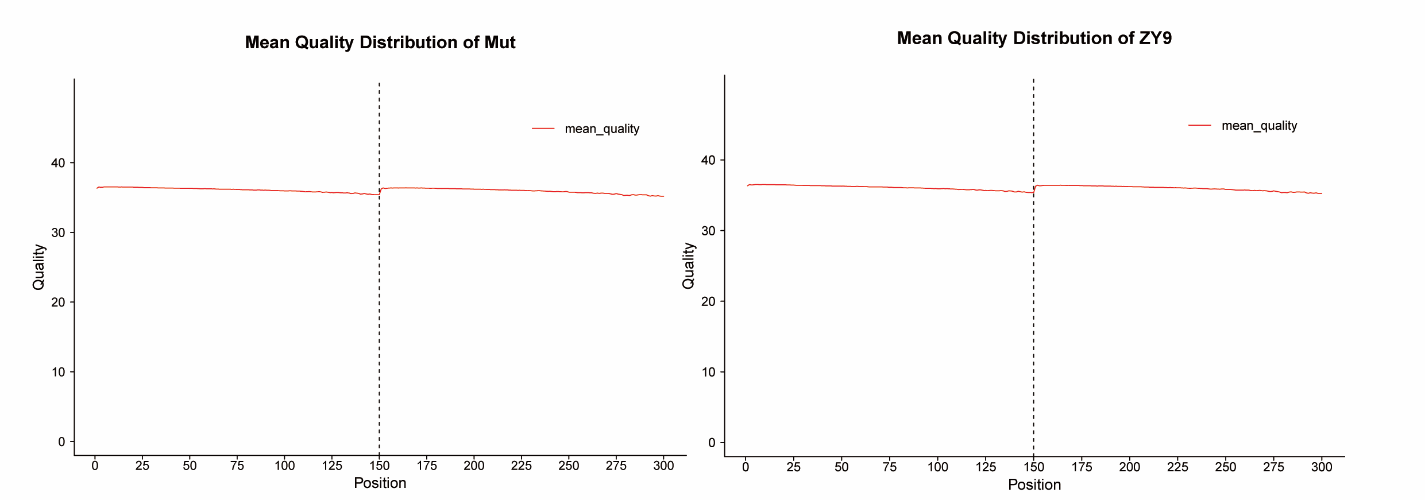


Figure S1 Distribution of sequencing Phred score in *eh1* (Mut) and WT (ZY9). The X-axis represents the position of bases in pair-end sequencing reads and Y-axis shows the average quality score of each base.


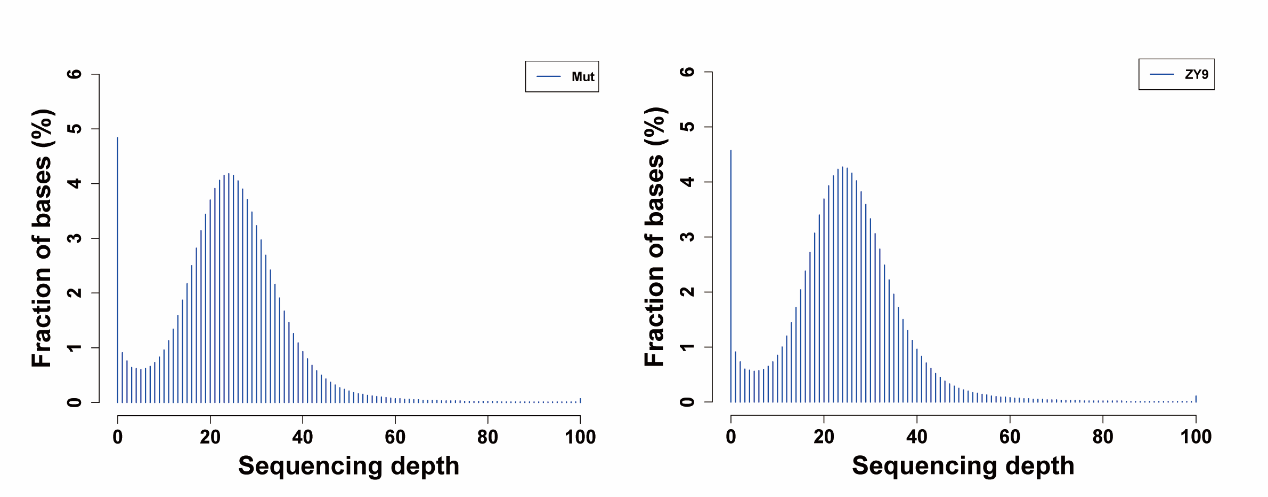


Figure S2 Distribution of base sequencing depth in *eh1* (Mut) and WT (ZY9). The Fraction of bases represents the number of bases at a given depth divided by the length of whole genome, which reflects the genome coverage at a given depth.


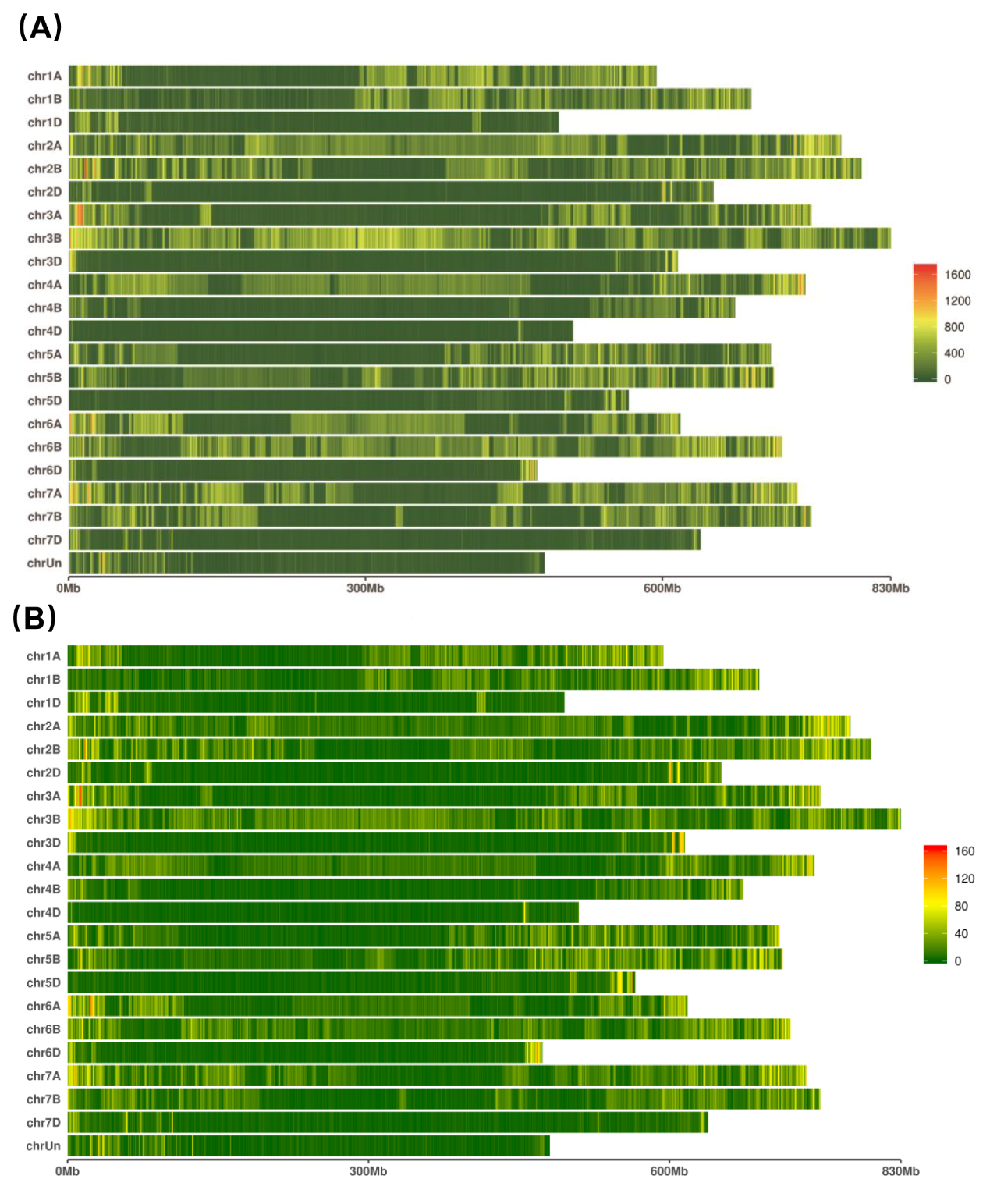


Figure S3 Distribution of exonic variations on all chromosomes. The distribution of (A) SNP and (B) Indel identified by exome-capturing sequencing. Ranges of color indicate the density of variations. The plot was visualized by CMplot package.


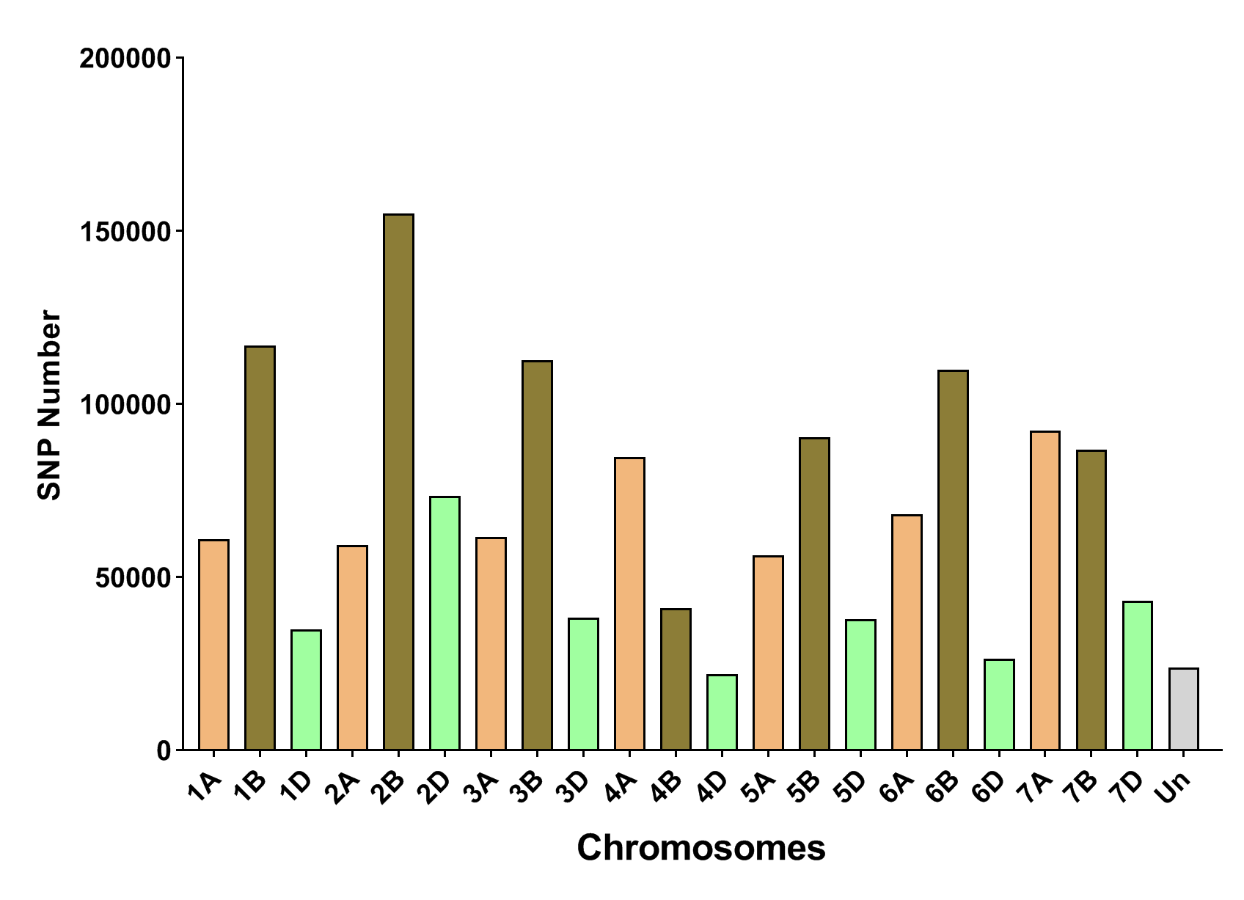


Figure S4 Distribution of SNP number identified in 12 gamma-ray-induced wheat mutant lines.


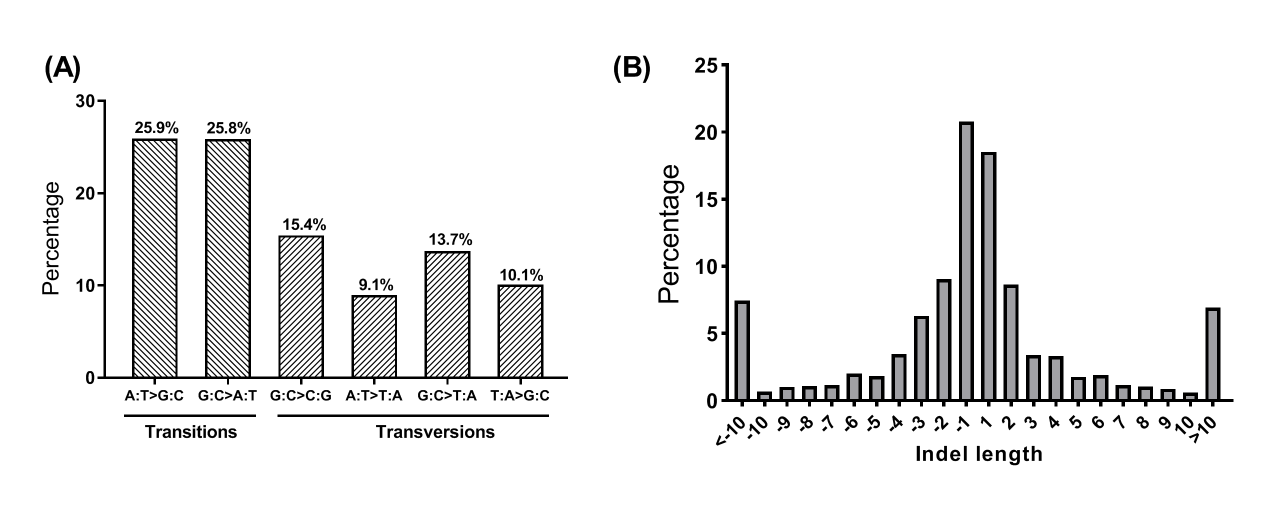


Figure S5 Variation types of exomic SNP and Indel in 12 gamma-ray-induced mutant lines. (A), The fraction of specific types of SNP in the exome of all mutant lines. (B), Statistics of the size of Indel identified by exome-capturing sequencing. The positive and negative value represents insertions and deletions, respectively.


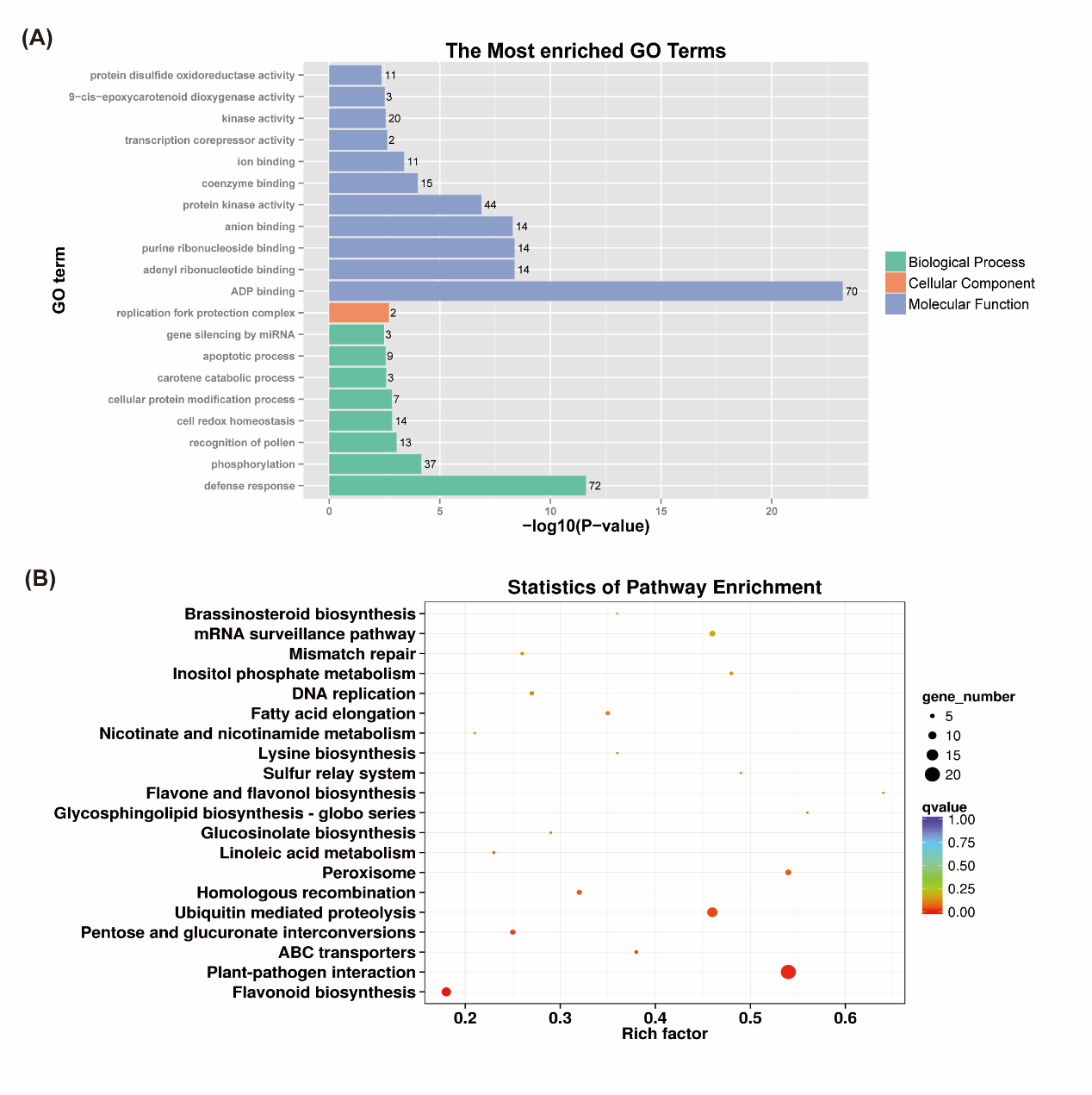


Figure S6 GO and KEGG enrichment analysis of functionally mutated genes in 12 gamma-ray-induced mutant lines. (A), The top 20 GO terms enriched in genes with termination or frameshift variations. The significance of enriched terms were determined by REVIGO. (B), The most enriched KEGG pathways in genes with termination or frameshift variations. The rich factor represents the degree of mutated genes in a given pathway. The number of enriched genes is indicated by the size of the circle and the circle color reflects the range of corrected P value.
