# Supplementary material for: Genome-Wide and Exome-Capturing Sequencing of a Gamma-Ray-Induced Mutant Reveals Biased Variations in Common Wheat
Source: Front Plant Sci. 2022 Jan 12;12:793496. doi: 10.3389/fpls.2021.793496 (PMC8790116; doi:10.3389/fpls.2021.793496)
Supplement: Supplementary file 2 [file Data_Sheet_2.docx]

Table S1 Distribution of SNP numbers identified by exome-capturing sequencing in 12 gamma-ray-induced mutants

Table S2 Primer sequences used in KASP assay

| Genes | Chr. | Position | Poly. | Forward Primer (5’-3’) | Rverse Primer (5’-3’) |
| --- | --- | --- | --- | --- | --- |
| *Glu-A1* | 1A | 508723664 | Yes | TTCCCTTTCCCTTGGAGGC**T/C** | TGGCAATATCCCTGGCTAAAACTAT |
| *Glu-A1* | 1A | 508724101 | Yes | ACCTTTAGGCTGGCCGC**A/C** | ACAAGGGCAACAATTAGGACAA |
| *Glu-A1* | 1A | 508725730 | Yes | ATTGCAACAACCAACACAAG**A/G** | TAGTTGCTGCCCTTGTCCTG |
| *Glu-A1* | 1A | 508725794 | Yes | GTTGGATAGTACCCTGGTTGCT**T/C** | CCAACTTCTCCGCAGCAGT |
| *Glu-A1* | 1A | 508725967 | Yes | TGGCCTGGATAGTATGAAACC**T/C** | CGAGACAATATGAGCAGCAAGTC |
| *Glu-D1* | 1D | 412161151 | Yes | ATCGGGACAAGGGCAACAT**G/T** | CTGACAGCTGCGGAGAAGTT |
| *Glu-D1* | 1D | 412162959 | Yes | GTATGAAACCTGCTGCGGA**G/C** | GCAGCAACTCCAACAACGTA |
| *Ppd-A1* | 2A | 36933891 | Yes | GGCTTTCGGAAAAACCCAG**T/C** | GTGGCCGCCGTGAACAAG |
| *Ppd-A1* | 2A | 36934685 | Yes | CCGTCTCACAACGTTCCTC**T/C** | AGATCGACCGCAGACGAC |
| *TaMFT-B1* | 3B | 4744301 | Yes | CCACCACCTCACCTGCA**T/C** | TGGATAATACGTCCGGGTGTA |
| *Fhb-B1* | 3B | 8528973 | Yes | GTCTGAGGACCGGTGGC**G/A** | GTCAACCAGGAAGGACCATT |
| *Wx-A1* | 4A | 688097944 | Yes | CCCGGTCTTGCCCTCCA**T/C** | ATGACCGAAGTTTCTTTCAAATTTG |
| *Psy-A1* | 7A | 729399643 | Yes | ACATCTTCAAAGGAGTCGTCA**C/G** | TTGATCTGCCTCTTCATGAATTTT |
| *Psy-A1* | 7A | 729400849 | Yes | GTTTCGTTTGTCGGCCTTT**G/T** | CCCACTATAGCGTCTAGTCAACA |
| *VRN-A3* | 7A | 71670910 | Yes | ATAATTTGCTGACTTTGCGGG**C/T** | ATTGACCTACCCATGGCCCA |
| *Psy-B1* | 7B | 739444007 | Yes | AATTGTTCAGTCCACATTGTATGA**T/C** | TGAGATTGTGAGGTCTAGGGA |
| *TaGS-D1* | 7D | 6484973 | Yes | TCAATCACCTCCATATGTCATGAA**C/T** | TTCATGTGTCAATCAGTTCACAC |
| *TaGS-D1* | 7D | 6485222 | Yes | CAACATATATTAAGATCTGCAGGG**A/C** | TGATCAATCTCATCGCTTCCCA |
| *Glu-D1* | 1D | 412162768 | No | CCAACTTCTCCGCAGCAG**C/T** | TGCCCTTGTGCTGGTTGTT |
| *Fhb-B1* | 3B | 8529230 | No | CACGAGTTGCTTCCCGTC**A/T** | GTCTCACAAAAGCCGGCAT |
| *Fhb-B1* | 3B | 8529304 | No | TTCACTGGACTCTGTGACG**G/T** | GCAGAGCCGGAGACAGTAAC |
| *Glu-A1* | 1A | 508723770 | Unknown | GGATATTGCCACACCCTCTTT**T/C** | ACAATAAATGTGGCGTGTGTTCAA |
| *Ppd-A1* | 2A | 36937810 | Unknown | GGCTCGGTCGTCATGGA**G/C** | CATGACACACAACCAACGCC |
| *TaMFT-B1* | 3B | 4744195 | Unknown | CGCTTCCCCTACGTCGC**G/C** | CCGGACGGCTCCTTCTGA |
| *Fhb-B1* | 3B | 8529306 | Unknown | AACTGGGGCAAGCAAACAT**T/G** | GCCTCAGAACCTGATTGGCA |
| *Psy-B1* | 7B | 739444091 | Unknown | GGTGATGGTGTCGGAGAG**C/T** | GACGAGCTGGTGGACGGG |

Table S3 Identification and annotation of variations in major flowering genes
